# New Structural insights into Kir channel gating from molecular simulations, HDX-MS and functional studies

**DOI:** 10.1038/s41598-020-65246-z

**Published:** 2020-05-21

**Authors:** Charline Fagnen, Ludovic Bannwarth, Iman Oubella, Eric Forest, Rita De Zorzi, Aline de Araujo, Yasmina Mhoumadi, Saïd Bendahhou, David Perahia, Catherine Vénien-Bryan

**Affiliations:** 1 Sorbonne Université, UMR 7590, CNRS, Muséum National d’Histoire Naturelle, Institut de Minéralogie, Physique des Matériaux et Cosmochimie, IMPMC, 75005 Paris, France; 2 Laboratoire de Biologie et de Pharmacologie Appliquée, Ecole Normale Supérieure Paris-Saclay, Centre National de la Recherche Scientifique, 91190 Gif-sur-Yvette, France; 3 University Grenoble Alpes, IBS, F-38044 Grenoble, France, CNRS, IBS, F-38044 Grenoble, France, CEA,IBS, F-38044 Grenoble, France; 4 Department of Chemical and Pharmaceutical Sciences, University of Trieste, Via Licio Giorgeri 1, 34127 Trieste, Italy; 5 CNRS UMR7370, LP2M, Université Côte d’Azur, Faculté de Médecine, Nice, France

**Keywords:** Mass spectrometry, Molecular modelling

## Abstract

Inward rectifier potassium (Kir) channels play diverse and important roles in shaping action potentials in biological membranes. An increasing number of diseases are now known to be directly associated with abnormal Kir function. However, the gating of Kir still remains unknown. To increase our understanding of its gating mechanism, a dynamical view of the entire channel is essential. Here the gating activation was studied using a recent developped in silico method, MDeNM, which combines normal mode analysis and molecular dynamics simulations that showed for the very first time the importance of interrelated collective and localized conformational movements. In particular, we highlighted the role played by concerted movements of the different regions throughout the entire protein, such as the cytoplasmic and transmembrane domains and the slide helices. In addition, the HDX-MS analysis achieved in these studies provided a comprehensive and detailed view of the dynamics associated with open/closed transition of the Kir channel in coherence with the theoretical results. MDeNM gives access to the probability of the different opening states that are in agreement with our electrophysiological experiments. The investigations presented in this article are important to remedy dysfunctional channels and are of interest for designing new pharmacological compounds.

## Introduction

**Figure 1:**
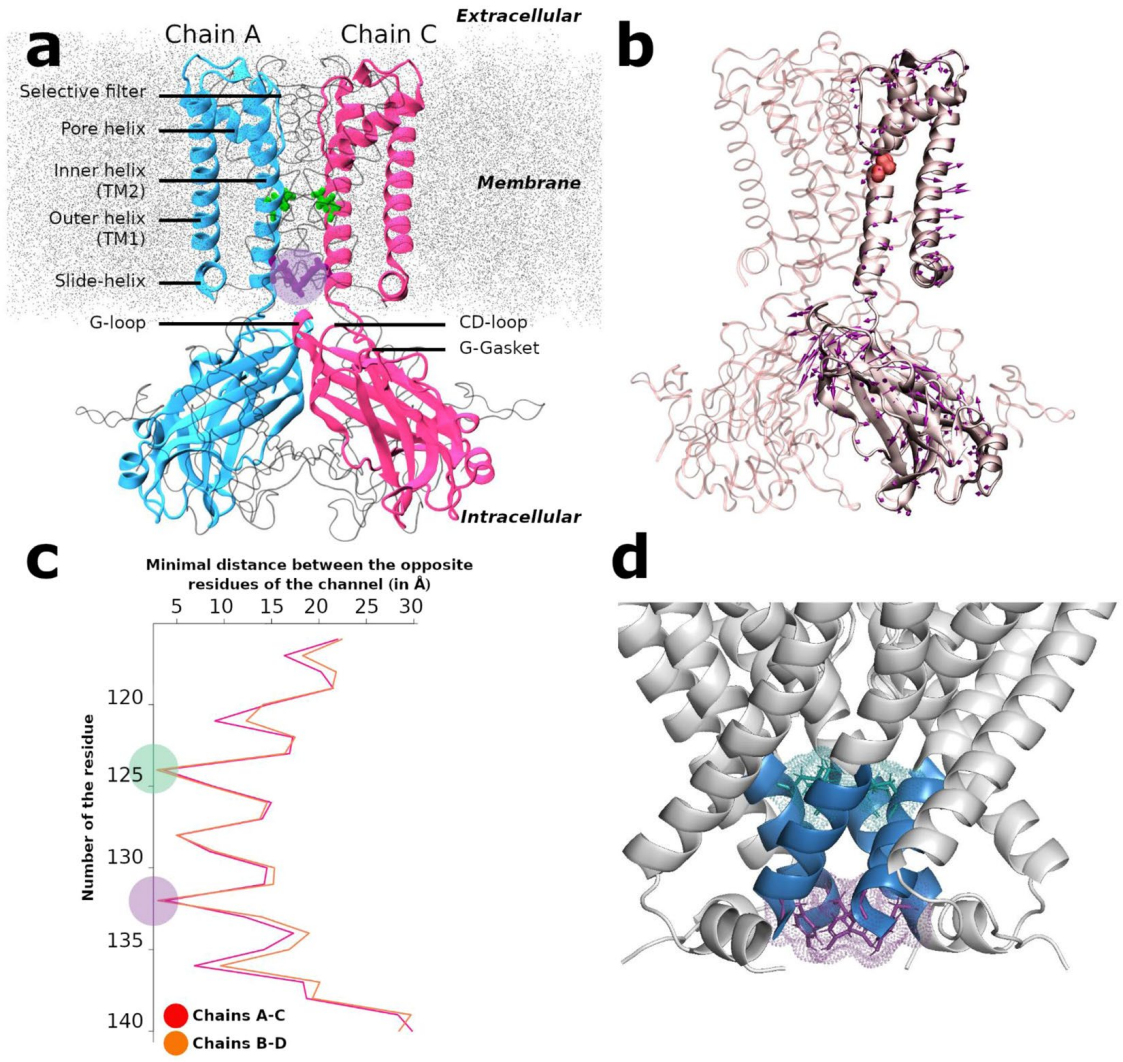
Structure, Normal Mode and constriction points of KirBac3.1. (**a**) Overview of the KirBac3.1 structure (from PDB 2WLJ). NewCartoon representation of two opposite monomers (chain A in blue, chain C in pink) for clarity, the other two subunits (chains B and D) are represented in Newribbon gray. Leu124 and Tyr132 at the constriction points are respectively in green and purple. The membrane is in dotted gray. The purple circle represents the Helix Bundle Crossing (HBC) gate. (**b**) Direction of motion of mode 123 involved in the opening/closing of the KirBac3.1 channel. Gly120 is in red in Van der Waals representation, arrows show the direction followed by the Cα atoms. (**c**) Minimal atomic distances on the plane parallel to the membrane between opposite residues of chains A and C (in red) and between chains B and D (in orange) versus residue numberings. The position of the constriction regions located at Leu124 and Tyr132 are highlighted in green and purple circles, respectively. (**d**) Crystallographic structure of the KirBac3.1 channel. Stretch of residues from Met121 to Ala 133 is in blue. The constriction residues Leu124 (green) and Tyr132 (purple) are highlighted.

Inward rectifier potassium (Kir) channels play diverse and important roles in shaping action potentials of cardiac myocytes, membrane potentials of nerve and glial cells, hormone secretion of pancreatic β cells and potassium homeostasis of kidney^1^. Kir Channel dysfunction causes an increasing number of diseases^1^. The flow of K^+^ ions through the pore is controlled by conformational changes in the Kir channels^2,3^ described as gating. KirBac3.1 is a prokaryotic Kir homolog (28% identity with human Kir2.1) that has the same characteristic structural elements as eukaryotic Kir channels^4,5^ (see also sequence alignment Fig. S1). The X-ray crystallographic structures reveal that KirBac3.1 is a tetramer and the secondary structural elements include the slide, outer (TM1) and inner (TM2) helices plus the pore helix, the selectivity filter, and the C-terminal cytoplasmic domain (CTD) (see Fig. 1a for nomenclature).

Different Kir-channel structures are available of both prokaryotic and eukaryotic channels, and those of the same channel in different conformational gating states are particularly informative. These structures have given some clues regarding the dynamics of the channel, for instance: (i) the kink in the TM1 and TM2 helices^5–7^ (ii) the slide helix orientation^7,8^ (iii) the cytoplasmic domain position relative to the transmembrane domain^9–11^ (iv) the K-channel selectivity filter described rather as a rather rigid entity^12,13^. However, these crystallographic “snapshots” might not be relevant to the dynamic gating conformations of ion channels as they give a partial and limited view of the process. One of the major challenges in ion channel structural biology is to understand how these different static crystallographic states relate to the function of the channel.

Targeted global structural rearrangements during gating have been investigated using single-molecule FRET measurements^14,15^ and structural mass spectrometry^16^. Despite valuable experimental data on structural dynamics of the channel^17^, the overall conformational changes of gating transitions remain speculative, and the global and local motions of the whole protein are still missing. Molecular dynamics studies have also been performed^18,19^, but the short simulation times (hundreds of ns) often make it difficult to observe the whole gating process.

In the present work, for the very first time, we provide a clear view of the transition from closed to open of the whole KirBac3.1 protein using *in silico* methods and experiments. We present a clear description of the global and local (side chain) motions involved in the gating mechanism, bridging the gap between the crystal structures and the dynamics of the channel. One of the main challenges in computational biology is to structurally characterize functional motions occurring at long time scales, in particular when large movements are involved such as in the gating mechanism of the Kir channels. To overcome this problem, we used a recently developed computational method: Molecular Dynamics with excited Normal Modes (MDeNM)^20^. The great advantage of this hybrid simulation is that it exhaustively explores the energetically accessible conformations within the space spanned by a given number of low-frequency normal modes, all of which are associated to slow collective dynamics of the molecular system. Moreover, MDeNM enables the exploration of relatively higher energy low-populated states that might be important to biological functions. We describe how different structural changes, such as the kinks of the internal and external TM helices, are associated to motions of slide helices and CTD (swinging and rotational movements) in the gating mechanism, at the global and local levels. Furthermore, the calculations provide open and closed states populations that can be compared with those obtained by electrophysiological experiments*. In silico* results are also supported by Hydrogen/Deuterium exchange coupled to mass spectrometry (HDX-MS). Different types of motions were involved in the gating, which appears more complex than currently perceived.

## Results

### Theoretical data

The theoretical results presented in this section are based on MDeNM simulations in which a selected set of normal modes (NMs) related to opening/closing of the channel are excited within molecular dynamics (MD) simulations. Through a combination of MD and NMs, the MDeNM is a realistic exploration of the relevant normal mode space for opening/closing processes taking into account the full environment (membrane, water, ions) of the protein at a given temperature. These simulations are completed by standard MD simulations carried out for a uniformly distributed set of structures that provide a fairly good estimation of the populations of the open/closed (and partially open) states.

#### Channel constriction points

To be able to select relevant NMs for gating, the positions of constriction points along the channel in the closed conformation crystallographic structure (2WLJ) were identified by scanning the minimal distances between facing residues along the segment forming the channel and buried within the membrane, i.e. between the residues 115 and 140. The width of the pore at each residue can be estimated using this method; the resulting values are given in Fig. 1c. The two most constricted regions correspond to the position of Leu124 and Tyr132 residues, with pore width minimal distances of 3.58 Å (Leu124AC: between opposite chains A and C) and 2.89 Å (Tyr132BD: between opposite chains B and D) respectively as shown in Fig. 1d. These distances are not sufficient to let a potassium ion with an ionic diameter of 3.53 Å^21^ hardly go through the channel unless global and local motions cause an increase of the pore diameter.

#### Selection of modes contributing to the gating

Eleven most representative modes out of 200 involved in the opening of the channel were identified (see Supplementary data). In one of the selected modes, shown in Fig. 1b, the arrows of motion in the regions under and above the residue Gly120 of the inner helix (red residue in Fig. 1b**)** are directed in opposite directions, accounting for the kinking of the inner helix observed in the X-ray crystallographic structure of KirBac3.1, open conformation^9^. A similar trend is observed for the external helix, in which the arrows indicate also a motion causing a kink at Ala62 facing Gly120. The bending of the outer helix is confirmed by rearrangements observed in cryo-electron microscopy (cryo-EM) data^6^. Both helices display a concerted motion during their kinking. In this type of collective motion, the lower half of TM2 helices move outward, contributing to the opening of the channel in the constriction regions. More interestingly, arrows on the slide helix indicate that its outward movement is correlated with that of TM helices, most probably contributing to the opening of the channel. As far as the CTD is concerned a swing type motion is observed. Note that Fig. 1b shows one particular mode where the opening of the channel has been investigated and the movement of the CTD is less important. Other modes will show different dynamics. The amplitude of the arrows are such that they correspond to a 1 Å of RMSD displacement and is just illustrative of the kind of movement. The final real amplitudes are those obtained by the cumulative contribution of all the selected modes provided by MDeNM simulations.

#### Population of open and closed states from the relaxed structures issued from MDeNM simulations

In order to get dynamic information from the selected normal modes described in Materials and Methods, we carried out MD simulation including the full environment (water, lipids, ions) using MDeNM. Four channel-gating states can be discriminated based on the open or closed conformation of the two main constriction points (Leu124 and Tyr132) observed in the ensemble of relaxed structures: 1) Fully open state, when the minimal distances at both the constriction points are greater than the ionic diameter of K^+^; 2) Fully closed state, when both distances are less than the ionic diameter of K^+^; 3) Half-open state 1, when gating at Leu124 is open and gating at Tyr132 is closed; 4) Half-open state 2, when gating Tyr132 is open and gating at Leu124 is closed. A clear definition of open or closed functional states is challenging to give in the context of our study. We have given a minimum definition based on the distances between the opposing residues at constriction points, which could provide an estimate on the minimal probabilities of having the open state. Inevitably, we overlook other possible effects such as ion permeation through a channel with a diameter smaller than the size of the ion as happens along the protein selectivity filter. Such permeation implies an induced effect of ions favoring structural adjustments as they pass through the channel, which this study cannot address. Rather, our objective was to show how global movements could participate in channel widening that could increase the probability of ion flux. Our definition of open and closed states is supported by the representation of fully closed, fully open and half open structures with the HOLE software **(**Fig. S2**)**. It is clear that in our closed structure the conduction pathway is shut (red patches at the two constriction points of the channel) and that the open structure can accommodate water molecules in the channel and thus K^+^ ions. Also, we checked the open structure’s ability to accept a polyamine. Under physiological conditions, the inward rectifying current from the Kir channels are the results of intracellular block of the polyamines. A spermine molecule was docked in one of our KirBac3.1 WT open structures with the Autodock Vina software (Fig. S3). The open state structure of the channel allows the positioning of the spermine so that the state of the structure can be considered as open.

Interestingly, the fully open state is only populated of about 6.8%, and it is worth noticing that the gating at Tyr132, closer to the cytoplasmic domain, is more often closed and more restricted than the gating at Leu124 (28.8% compared with 14.2%, respectively, Table 1). These data are consistent with our experimental functional data (see below).

**Table 1:** Populations (in percentage) of different opening states in the relaxed structures of KirBac3.1 WT obtained through MDeNM simulations.

Channel States	KirBac3.1 WT
Fully open	6.8%
Fully closed	50.2%
Half open 1 (124 open, 132 closed)	28.8%
Half open 2 (132 open, 124 closed)	14.2%

**Figure 2:**
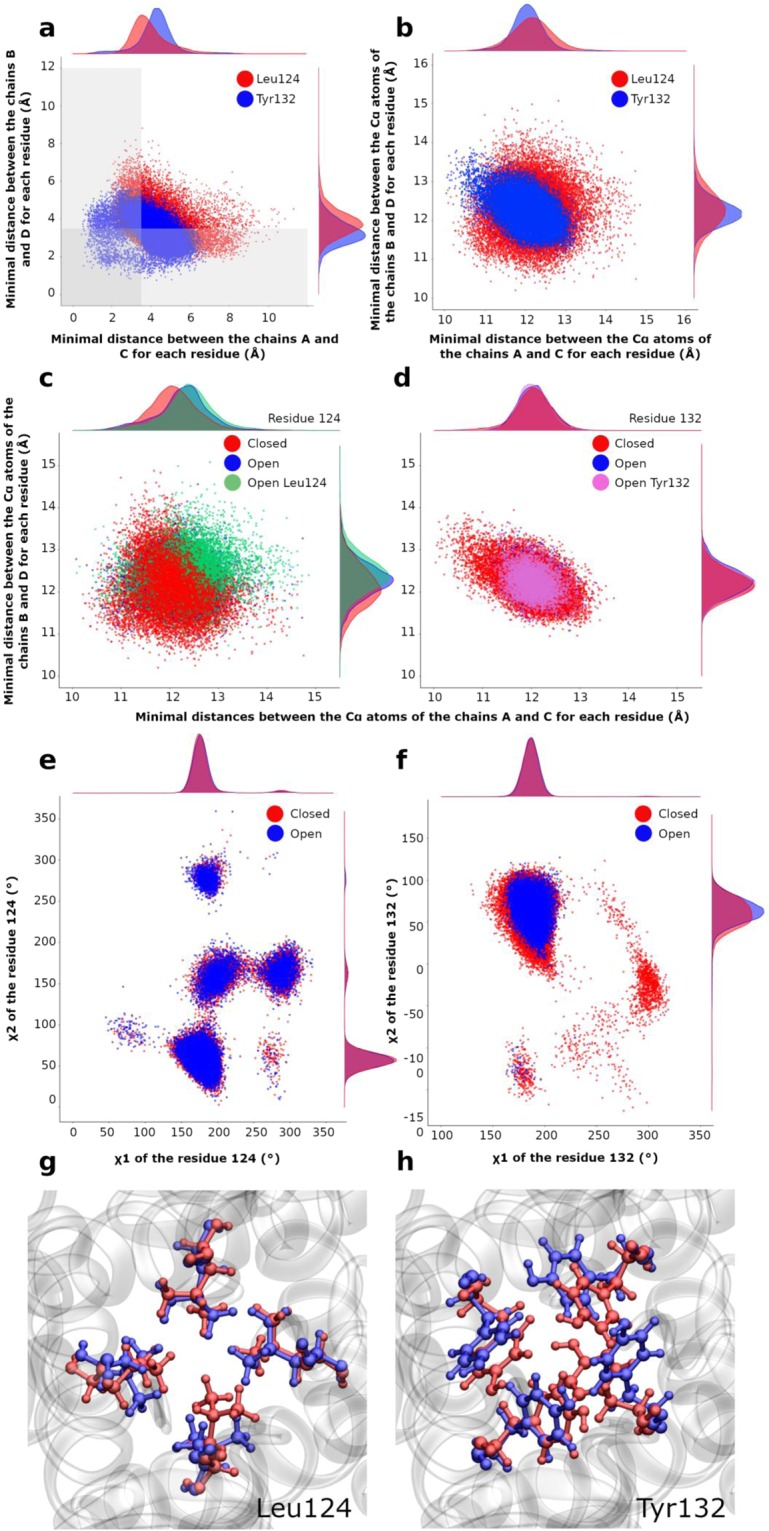
Gating at the two constriction points Leu124 and Tyr132. (**a**) Scatter plots of the minimal distances between the chains B and D and between A and C. Red and blue points correspond to the residues Leu124 and Tyr132, respectively. The gray area delimits the region where the channel is closed. (**b**) Scatter plots of distances between Cα atoms of the residues 124 (in red) and 132 (in blue) for chains AC and BD. (c-d) Scatter plots of Cα-Cα distances on X-Y plane between chains A and C (abscissa) and between chains B and D (ordinate) at the residue Leu124 (**c**), and at the residue Tyr132 (**d**). Each point is colored in function of its state: red for closed, blue for open, green for only open for the Leu124, purple for only open for Tyr132. Along each axis, the population density curve of the points is given for each state of the channel; (**e-f**) Scatter plots of χ1 and χ2 angles for all the chains for Leu124 (**e**) and Tyr132 (**f**); (**g**) Superposition of the most open structure (in blue) with the most closed structure (in red) for residue Leu124; (h) same as in (g) but for residue Tyr132.

More details about the gating states can be obtained by analyzing the scatter plots of minimal distances between the opposite chains A and C and those between chains B and D at both constriction points over all the MDeNM relaxed structures as shown in Fig. 2a. Minimal distances between the chains A and C (AC) at Leu124 range from 1.52 to 11.30 Å, and between the chains B and D (BD) from 1.66 to 8.81 Å (Fig. 2a in red). The corresponding values at Tyr132 range from 0.44 to 7.00 Å and from 0.75 to 6.22 Å, respectively for chains AC and for chains BD (Fig. 2a in blue).

From the gray areas in Fig. 2a, it is clear that structures with a closed gate at Tyr132 are much more populated (more than 79.00% of all the structures) than those with a closed gate at Leu124 (around 64.40%). Therefore, Tyr132 constitutes the main constriction point. Interestingly, from a statistical point of view, one can observe that larger distances between the chains A and C correspond to smaller distances between chains B and D, and vice versa, indicating an elliptical movement of the channel.

#### Implication of global and local motions to the gating

MDeNM simulations couple global and local motions through NM excitations in the framework of MD simulations, making it possible to distinguish the implication of both types of motions in the gating. To determine the contribution of the global motions to channel opening/closing, the scatter plot of Cα-Cα distances between opposite residues in the AC and BD chains were analyzed at the constriction points level. This was done over the same relaxed ensemble as used for the minimal distances. The plot shape for distances between Cα atoms at Tyr132 (Fig. 2b, in blue) is similar to that of minimal distances between chains (Fig. 2a in blue), suggesting that global motions are playing an important role in the opening of this gate. Nevertheless, in the minimal distances plot, some points are scattered outside the elliptical core, towards smaller values (Fig. 2a), indicating the contribution of local motions too. The same analysis at Leu124 (Fig. 2a,b in red) shows a larger outgoing dispersion of minimal distances towards the closed region, indicating that the local motions play a significant role in the opening/closing of this gate of the channel.

In order to better differentiate the implication of global and local motions, different subsets of structures were considered: a subset of structures closed at both constriction locations (Leu124 and Tyr132), a subset fully open in both locations, and two subsets of structures open in one site and closed in the other. The Cα-Cα distance scatter plots between opposite chains AC and BD of all these subsets are given in Fig. 2c-d for both constriction regions.

Some general features can be noted: (1) the distributions are in general more circular at Leu124 (Fig. 2c), and more elliptical at Tyr132 (Fig. 2d), which is indicative of an inverse correlation between distance variations of chains AC and BD at Tyr132 for either open and closed states; (2) The closed states for both constriction points are centered at Cα-Cα distances of about 12 Å between facing chains; (3) A larger variation in the distribution of points is obtained at the constriction of Leu124 rather than at Tyr132 indicating a larger global structural variability at Leu124, consistent with the fact that Leu124 gating is less restrictive.

A more detailed analysis points out that at Leu124 (Fig. 2c) both the AC and BD Cα-Cα distances corresponding to the open state (blue and green) have their maximal populations located very close to each other, but are further away from the most populated distance of the closed state (red). Furthermore, when AC and BD distances are simultaneously larger than 12.8 Å, the structures are mostly open, considering that such value is too large for local motions of residues to be able to close the channel. For distances below 11.0 Å, the region is highly restricted to the point that no side chain movement would allow an opening of the channel. For distances between 11.0 Å and 12.8 Å, local motions have a determinant role: opening/closing of the channel in this range of distances depends only on motions of side chains. There is interplay between the global and local motions for the opening/closing of the gate at Leu124, the former determining the widening range of the channel, and the latter intervening mostly within a given intermediate range.

As far as the gate at Tyr132 is concerned (Fig. 2d), the population distributions of open and closed states are very close to each other. However, extreme distances between the Cα atoms of chains AC, or between chains BD, such as below 11 Å or above 13 Å, correspond mostly to closed states, depending solely on global motions. Note that due to the elliptical shape of the scatter plot at Tyr132 an increase in the distance between two opposite chains is associated with a decrease in the distance between the other two chains. The opening of the channel requires that the minimal distance between the Cα atoms of opposite chains be in a certain range so that the side chain motions can intervene for opening and closing the channel (between 11 Å and 13 Å).

#### Side-chains rotational motions of residues at the constriction regions associated to gating

In order to better highlight the importance of side chain motions in the opening/closing of the channel at both constriction points, we analyze here the distribution of side chain rotational angles (χ1 and χ2) in the various open and closed subsets (Figs. 2e,f**)**. For Leu124, all rotational conformations occur either in the open (blue) or closed (red) states with large variations of χ1 and χ2 angles, between 50° and 350°. χ2 adopts all four isomeric rotational values, the most favored (χ1,χ2) combinations being (180°,60°), (180°,160°), (180°,280°), and (280°,160°) **(**Fig. 2e). However, a different rotational state in only one given chain could be sufficient to let K^+^ ions to go through the channel (Fig. 2g).

The (χ1, χ2) values of Tyr132 (Fig. 2f**)** display a major group around (200°,75°) either in the closed or open states, and two minor ones around (180°,−150°) and (300°,−40°) in the closed state. In the major group slight variations of χ1 and χ2 rotations can be crucial for opening/closing of the channel without excluding the global motions as illustrated in the Fig. 2h.

#### Correlations between types of global motions and gating from the relaxed structures obtained following MDeNM simulations

**Figure 3:**
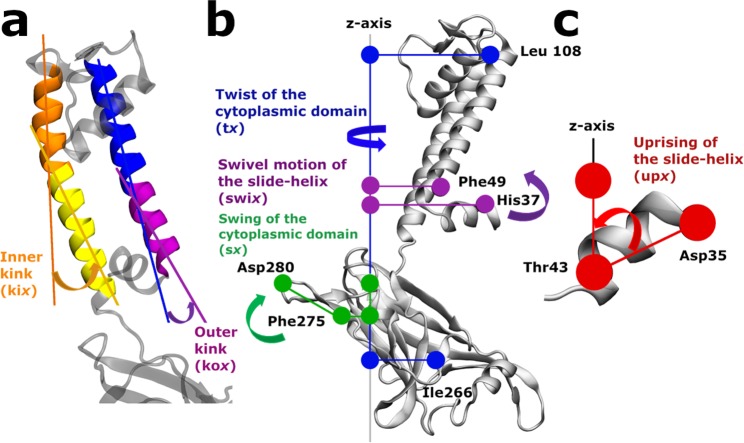
Description of the structural determinants. (**a**) Kink angle of the inner helix (*kix*) defined by the two axes: Ala109 to Gly120 (in orange) and Gly120 to Phe135 (in yellow). Kink angle of the outer helices (*kox*) defined by the two axes: Cys71 to Leu56 (in blue) and Leu56 to Trp46 (in purple) (**b**) Swivel angle of the slide-helices (*swix*) in purple, the twist angle (*tx*) in blue and the swing angle *sx* in green of the cytoplasmic domain. (**c**) Uprising angle of the slide-helices (*upx*). *x* character in the naming of structure determinant stands for the chains A, B, C and D. (see supplementary data for the definition of the angles).

**Figure 4:**
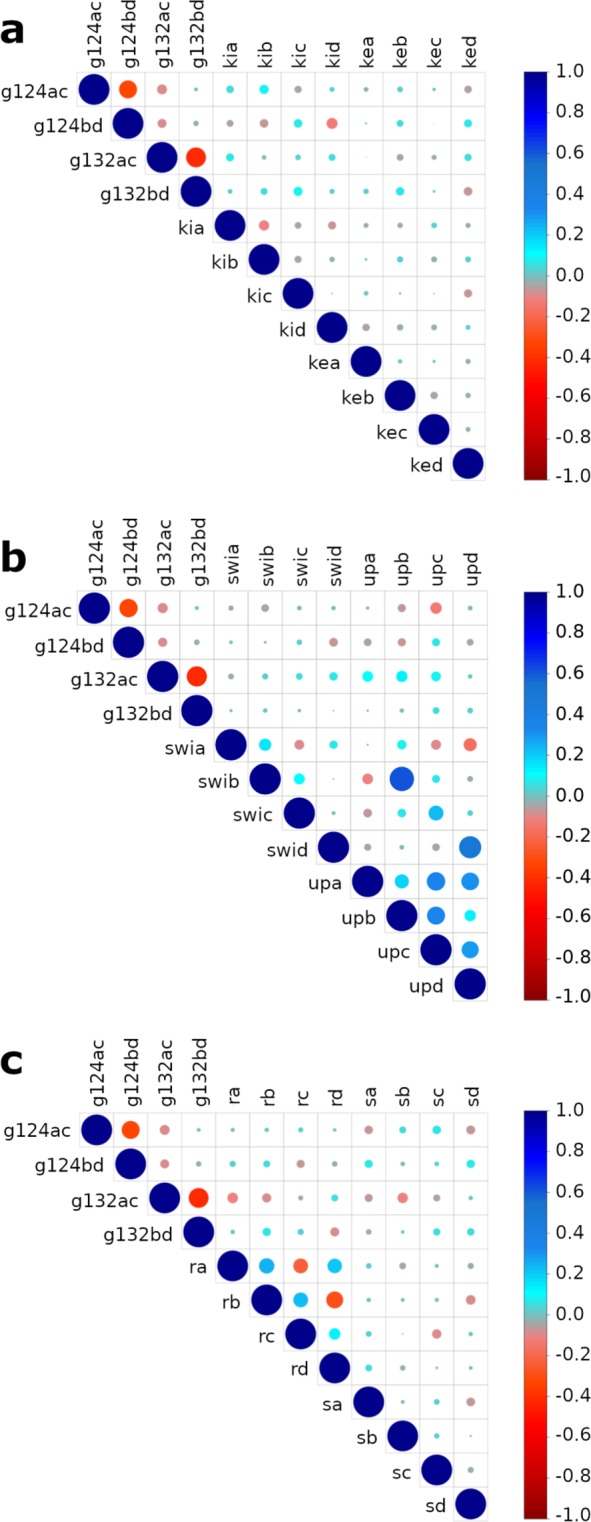
Graphs of normalized correlations between the structural determinants within the ensemble of MDeNM relaxed structures. g124ac, and g124bd are the minimal distances at the residue Leu124 between the opposite chains A and C and between the chains B and D, respectively; g132ac and g132bd are those relative to the constriction point at residue Tyr132. The area of the circle is proportional to the correlation strength between two parameters, the blue color indicates a positive correlation, and the red one a negative one (inverse correlation). The correlation value is given by the color scale bar at the right of the figure. (**a**) Correlations between minimal distances between the chains at the constriction points and the kink angles of TM helices. kia, kib, kic, kid represent the kink of the inner helices of the chains A,B,C and D, respectively; koa, kob, koc, kod represent the kink of the outer helices of the chains A,B,C and D, respectively (**b**) Correlations between minimal distances between chains at constriction points, the swivel (swia, swib, swic, swid) and uprising angles (upa, upb, upc, upd) of the slide-helices; (**c**) Correlation between minimal distances between chains, the twist and swing angles of the cytoplasmic domain. ta, tb, tc, td represent the twist angles of the cytoplasmic domain of the chains A, B, C and D respectively. sa, sb, sc, sd represent the swing angles of the cytoplasmic domain of the chains A, B, C and D respectively.

Kink of the outer and inner helices. The correlations between kink angles of TM helices **(**Fig. 3a) and the minimal distances between them at the constriction points are presented in Fig. 4a. The gating (*g*) Leu124BD (*g12*4*bd*) is correlated with the kink angles of the outer TM1 helices at Leu56 (*kox*, where *x* stands for the chain) of chains B (*kob*) and D (*kod*); see Materials and Methods for definition for the definition of the structural determinants. The gating Tyr132AC (*g13*2*ac)* is correlated with the kink angles of the inner TM2 helices at Gly120 (*kix*) of chains A (*kia)* and D *(kid)* and the gating Tyr132BD (*g132bd)* is correlated with *kib* and *kic*.

These results highlight that the gating at Tyr132 is linked to the kink of the TM inner helices whereas the gating at Leu124 is linked to some extent to the kink of the outer TM helices

Slide-helices. The swivel (Fig. 3b, purple) and upward (Fig. 3c) motions of the slide helices are involved in the gating of the channel as shown in Fig. 4b. It may be observed that the swivel motion of the slide helices of chains B (*swib)* and D (*swid)* are inversely correlated with the minimal distance at Leu124AC (*g124ac)* and Leu124BD (*g124bd)* respectively. The swivel motions of the slide helices of chain C (*swic*) and D (*swid*) are correlated with the minimal distances at *g132ac*. The minimal distance at *g124bd* is correlated with the uprising motion of the slide-helix of chains C (*upc*). The simultaneous upward movement of all the slide-helices (*upa*, *upb*, *upc* and, more weakly, *upd*) favors the opening of *g132ac*. The upward movements of the slide helices in chains C (*upc)* and D (*upd*) are also correlated with *g132bd*.

Cytoplasmic domain. Figure 4c describes the normalized correlations between the opening of the channel and the twist or swing angles of the cytoplasmic domain (Fig. 3b blue and green respectively). The minimal distance at *g124bd* is correlated with the twist angle of the cytoplasmic domain of the chains A (*ta*) and B (*tb*), however, the minimal distance of *g132ac* is inversely correlated with *ta* and *tb*.

As far as the swing motions of the cytoplasmic domain are considered, the minimal distance at Leu124AC (*g124ac)* is inversely correlated with the swing angles of chains A (*sa)* and D (*sd*), but that of Leu124BD (*g124bd)* is correlated with *sa* and *sd*. The minimal distance at Tyr132AC (*g132ac)* is inversely correlated with the swing angle of chains A (*sa*) and B (*sb*), and that of Tyr132BD (*g132bd)* is correlated with the swing angles of chains C (*sc*) and D (*sd*). Overall the gating at Leu124AC is influenced when the swing motions of chains A and D are opposite to those of chains B and C, and the gating at Tyr132 is influenced when the swing motions of chains A and D are opposite to those of chains C and D.

### Experimental data

#### Hydrogen/Deuterium exchange coupled to mass spectrometry (HDX-MS)

Protein conformational dynamics of KirBac3.1 were investigated using HDX-MS, a method based on hydrogen/deuterium exchange at the amide backbone of a protein, reflecting its dynamical properties. While protein HDX occurs at any site containing labile hydrogen, amides on the peptide backbone are by far the most commonly measured exchanges in HDX experiments. HDX has been widely used on soluble proteins and also on membrane proteins^22,23^. HDX was performed on the purified KirBac3.1 protein in detergent. We have established in earlier work that the presence of detergent does not affect the conformational changes of KirBac channel^16^. To achieve sequence coverage as large as possible, we tested various proteases. Optimized conditions led to sequence coverage of 60% and 70% using pepsin in solution and on column respectively, and a larger coverage of 86% with nepenthesin (Table S1 and Fig. S4**)**. Deuterium incorporation was monitored as a function of time for each peptide generated from KirBac3.1 (Fig. S5).

**Figure 5:**
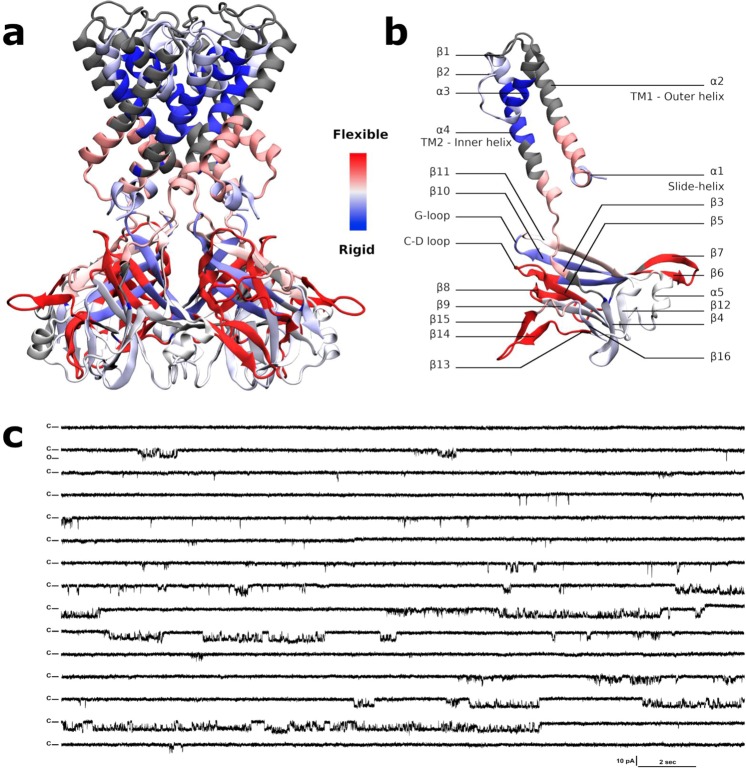
HDX rates of peptides reported on the KirBac3.1 model (2WLJ) and functional studies on the channel. (**a-b**) Identified peptides are drawn with blue to red color according to their percentage of deuterium exchange after 1,200 s (scale of exchange shown between panel a and panel b). Gray color is used in regions where no data are available. (**c**) Single channel recordings from wild-type KirBac3.1 channels; example of traces for 6-min consecutive recordings at −80 mV holding potential. Closed level is labeled C, and O for open.

The most flexible region of the protein **(**Fig. 5a**)** is located in the CTD, the stretch of residues (aa) 271–285, i.e. the loop between β14 and β15 with an HDX rate of 59.1% (for secondary elements numbering refer to Fig. 5b and Fig. S1**)**. The loop is subjected to the swinging movement taking place during the gating described earlier. The next, more flexible, domain is at the G gasket just below the CD-loop and the entire β8, (Fig. 5b, aa 199–210, HDX rate of 57.9%). A similar percentage (57.7%) is shown for i) the stretch going from the end of the β5 and the start of the β6 including the CD loop in the middle (aa 162–174), and also ii) the stretch going from the end of β6 and the start of β7 including the loop in the middle facing down (aa 181–194). The stretch including the end of the G loop and the following β11 also shows some flexibility (aa 250–260, HDX rate of 38%). As a general characteristic, the center of the cytoplasmic domain is more rigid (Fig. 5a, blue) than the outer surface of the cytoplasmic domain (in red).

In the transmembrane domain, the very end of the slide helix and the bottom half of the TM1 are clearly more flexible (aa 43–56, HDX rate of 45.2%), note that the top half of the TM1 is not accessible using this method. The stretch at the bottom of TM2-loop and β3 that includes the restriction point Tyr132 shows some flexibility (aa 132–143, HDX rate of 42.5%) unlike the top of the TM2, which is particularly rigid (Fig. 5a **in blue**). Note that the restriction point Leu124 is not detected in HDX-MS **(**Fig. S6). The slide helix shows some flexibility as well (aa 35–42 HDX rate of 21.4%).

#### Current recordings of KirBac3.1 in planar lipid bilayers

KirBac3.1 channels exhibit significant gating heterogeneity states as current recordings at −80 mV show (Fig. 5c**)**. Fits of Gaussian distributions of multiple histograms led to a single channel current level of 3.75 ± 0.027 pA at −80 mV corresponding to a 47 pS conductance (Fig. S7a). Analysis of all events of the full 6-min recording at this potential revealed the presence of multiple subconductance states of KirBac 3.1 (Fig. S7b). Subconductance states have also been reported for the KirBac 1.1^24^ and KirBac3.1^10^ channels. Interestingly, the calculated mean open probability (Po) at this potential was 9.9% (±1.3, n = 1,803). A close by value of (Po = 12%) was obtained for the KirBac1.1 channel^15^ supporting the theoretical opening value of the channel of about 6.8%.

## Discussion

In this paper, we examined the inherent motions of KirBac3.1, which are definitively associated with gating, i.e. the opening of constriction points of the channel, corresponding to residues Leu124 and Tyr132, with the constriction point at Tyr132 being more often closed. Theoretical data show an opening of the channel about 6.8%, this is supported by experimental data (Po = 9.9%) and also by the comparison with the value obtained for the KirBac1.1 channel^15^. Two main subconductance states (13 pS and 25 pS) were observed (corresponding to 1.02 and 2.03 pA current levels at −80 mV), Fig. S7b. The multi-conductance behavior of the KirBac channel has been attributed to ion interactions with the pore^24^ and could well explain several intermediate conformational states where the channel adopts two half open states.

The objective of this analysis was the study of the intrinsic movements of the channel in the absence of K^+^ within the channel. The passage of K^+^ ions through the channel depends on such movements, and the kinetics of the passage of the ion will certainly depend on its interaction with the pore and also to its hydration and dehydration states. As theoretical results carried out in the absence of K^+^ ions and experimental electrophysiological results accounting of these ions are very similar in the estimation of open state population. This fact tends to demonstrate that the intrinsic dynamical properties of the channel should play a major role in gating, and to suggest that the opening of the channel should not be mainly dependent on the presence of the K^+^ ions.

For the first time, we report here that local motions of residues in constriction regions, Leu124 and Tyr132, have a determinant role when the Cα–Cα distances of opposite chains are between 11 Å to 13 Å. Such distance variations depend on the overall global movements of the protein that were observed through HDX-MS experiments. In addition, simulation data show that the individual side chain motion of a single residue of Leu124 or Tyr132 in the tetramer, independently of the others, can be sufficient to open the channel and favor the passage of K^+^.

### Our studies show that there are multiple global motions that play an important role in gating as described below

#### Kinks of the TM helices

Observation of relaxed structures shows that TM2 (the internal helix) and interestingly TM1 (the outer helix) undergo kink variation up to 25.2° around the amino acid Gly120 and Leu56 respectively with standard deviation (SD) of 4.5° during the opening/closing process. A significant flexibility of TM2 is also shown with the HDX-MS experiments in agreement with previous crystallographic work^9^. The widening of the channel in another Kir protein, Kir3.2 is also associated with the rotation and splaying apart of the inner transmembrane helices as reported published *in silico* work^25,26^. Interestingly, the bottom of TM1 also shows flexibility as previously reported in published cryo-EM work (variation of the kink up to 20°)^6^. Note that this feature has not been observed in the crystallographic structure^9^.

#### Upward and swivel motion of the slide helix

This amphipathic slide helix is a ubiquitous structural motif of many K^+^ channels. It has been described as a “belt”^15^ around the channel on the cytoplasmic face of the membrane and has been proposed to be a key lining element between ligand-binding or voltage-sensing gating domains and the pore gating^27,28^. The slide helix shows a clear flexibility in our HDX measurements (Fig. 5B). More precisely, our theoretical data show that upon opening of the channel, the swivel (variation of 47.8° with a SD of 6.2°) and upward (variation of 21.4° with a SD of 3°) motions of this helix cause a movement toward the inside of the channel of the residue His37, at the extremity of the slide-helix (Fig. 4C). At the same time, Ser45, at the other end of the slide helix, moves towards the outside of the channel. A similar behavior of the four slide helices (strong correlation of the *upa, upb, upc* and *upd* parameters) demonstrates that the slide helices undergo a concerted upward movement during channel movement. Considering that the slide helices are not in direct contact with each other, it might be explained by the proximity of the membrane.

#### Swing motion associated with a twist of the cytoplasmic domain

Motions of the cytoplasmic domain that couple ligand binding to the gating of the channel have been thoroughly investigated and two different models have been proposed: The “upward motion model,” suggested by the structure of Kir2.2^11^, refers to an upward motion of the CTD, which translates towards and becomes tethered to the transmembrane domain. In addition, when the protein binds PIP_2,_ a small twist (5°) of the cytoplasmic domain is also involved in the gating^29^. Another model, the “twist to open” model proposes that a twist movement (approximately 25°) of the CTD relative to the plane of the membrane is crucial to allow gating^9^. However, work from another group has shown that the cytosolic domain is relatively independent from the pore-forming domain^10^. Single-molecule FRET has revealed the structural dynamics and flexibility of the CTD, with tilting or twisting motions of the CTD coupled to the HBC-gate closure^15^. Swing motion has also been supported by AFM experimental evidence^30^ and recent studies on GIRK2^26^. Our data show that the swing movement of the CTD region (variation of 121.6° with SD of 13.6°) is determinant for the opening of the channel. Twists (variation of 33.8° with SD of 5.1°) of individual chains can be associated with this swing movement. Our work indicate that the modes describing a full rotation of the CTD (excluding twist movement) were uncoupled with the gating of the channel (data not shown) as suggested by recent *in silico* studies^25,26^ and consistent with the X-ray data on GIRK2 bound to PIP_2_^11^. HDX results show that the center of the cytoplasmic domain is more rigid than regions at its surface; in particular, the external loop between β14 and β15 is highly flexible and accessible to the solvent. This evidence supports the swing movement of the cytoplasmic domain during gating. Another observation is that the motions involved in the gating mechanism often show an elliptical trend. This feature clearly indicates a behavior of the tetramer that does not respect the *C*_*4*_ symmetry of the crystal structure, but rather shows a *C*_*2*_ symmetric movement, such as a dimer of dimer behavior.

### Other movements make the channel fluctuate but are less important for the opening

Rotation of TM1 and TM2 around their own helix axis was observed, although not involved in the opening of the channel. Regarding the G-loop: our *in silico* study shows that it is very flexible. The minimum distance varies between 15 Å to 22 Å between the opposite chains A and C, and from 10 Å to 16 Å between the opposite chain B and D (Fig. S8). The flexibility of the G-loop has also been reported in molecular dynamics studies^25,26^ and X-ray data^31^. Interestingly our study shows that the distances between the opposite chains at this level (the distances between opposite residues from Glu248 to Ala251) are always greater than the diameter of the hydrated K^+^ and therefore the related region is not a gate. This finding is in contradiction with various published works^25,26,32^ which clearly show that the G-loop acts as a gate in eukaryotic Kir channel^33^ and is associated with the binding of PIP_2_^32^ and transduction. In the eukaryotic Kir channel, the key residues which are binding to PIP_2_ are located in two short linker regions that connect the transmembrane (TM) pore-forming region of the Kir channel to the large cytoplasmic domain. Each of these linkers is three residues long in the eukaryotic Kirs. Also, the second linker, between TM2 and the cytoplasmic domain, contains two charged residues which, when mutated, invariably cause loss of PIP_2_ activation and loss of PIP_2_ binding^34^. In contrast, prokaryotic KirBac channels lack these residues and is specifically inhibited by PIP_2_^24^.

Concerning the selectivity filter, the variation of the minimum distances between the opposite chains (residues Ile97 to Lys101) indicates certain fluctuations. The minimum distances vary from 5 Å to 9 Å between the opposite chain A and C, and from 3 Å to 8 Å between the opposite chains B and D (Fig. S9). However the link between these fluctuations and the gating of the channel was not apparent in our study. Other simulations studies have shown that the highest energy barriers to the movement of K^+^ ion are found in the selectivity filter^25^, in agreement with experimental data^16^.

## Materials and Methods

### Molecular modeling and dynamics

The simulations were carried out starting from the closed form of KirBac3.1 modeled from the structure with PDB code 2WLJ^10^ detailed at an atomic resolution of 2.6 Å. As the C-terminal and, more particularly, the N terminal segments of the protein were not fully resolved, the segments up to residue 34 and after residue 295 were ignored in the structural model considered in simulations. These regions of the protein are not likely to be involved in the gating process. Missing atoms were rebuilt using CHARMM program. The homotetramer was built using the “Protein Interface Surface and Assemblies” software through Pisa at EBI^35^; the ionization state of the protein residues was determined using PROPKA3^36,37^. The structure was oriented thanks to “Orientations of Proteins in Membranes” (OPM)^38^ software before its integration in a DOPC (1,2-dioleoyl-sn-glycero-3-phosphocholine) membrane in a box of 34,769 water molecules with 150 mM KCl built by the membrane builder module^39^ of CHARMM-GUI^40,41^. The box dimensions of the system are 116.2 × 116.8 × 133.3 Å^3^. TIP3P^42^ model was considered for water molecules together with the charmm36 force field^21^. We used periodic boundary conditions, and the PME algorithm to treat electrostatic interactions. The system was equilibrated during 600 ps at a constant temperature of 300 K using the Nose-Hoover thermostat and constant pressure of 1 atm using the Langevin piston. The Leapfrog Verlet algorithm was used for propagating the movement. The cut-on and cut-off distances for non-bonded interactions were set to 10 Å and 12 Å respectively, and between these values, the switching function was applied.

The MDeNM was carried out with CHARMM, and the free dynamics after MDeNM were carried out considering the same conditions but with NAMD^43^ v2.10.

### Normal modes

The protein complex at the end of equilibration MD was energy minimized in order to compute the lowest frequency NMs with all the atoms taken into account. For NM computation we used the CHARMM program with the Charmm36 force field. A switching function was applied to the non-bonded interactions; setting a cut-on distance of 10 Å and a cut-off of 12 Å. A distance dependent dielectric constant of 2r (r being the interatomic distance) was used. The minimization was carried out under harmonic restraints applied to atomic positions that were progressively decreased and finally continued without restraints by carrying out 50,000 steps of CG and continued with the ABNR algorithm using a convergence criterion of 10^–5^ Kcal mol^−1^ A^−2^ RMS energy gradient. 200 first lowest frequency modes ranged in ascending order of frequencies were computed using the iterative DIMB method^44,45^ within CHARMM.

### Selection of a subset of most contributing normal modes to channel gating

To proceed with the MDeNM calculations, we decreased the number of modes by selecting those that are mostly related to gating, i.e. contributing importantly to the channel diameter variations. This was achieved by structurally displacing the initial minimized structure of 2WLJ along each individual mode at an rms distance of 2 Å and by analyzing the structural changes of the channel region defined between the residues Met121 and Ala133, after superimposing them to this very region to compute a local rmsd. The larger local rmsd values were used as a criterion to detect the modes mostly affecting the region. We applied different filtering criteria to reduce the number of modes to a reasonable amount and to alleviate the combinatorial problem in MDeNM. The whole selection process resulted in 11 modes numbered 66, 74, 80, 114, 123, 135, 148, 169, 182, 192, and 197.

A visual inspection of the selected modes showed that they describe very satisfactorily the opening/closing of the channel, and can be considered as being very representative for the study of the channel dynamics. We give in Table S2 the Cα-Cα distance variations of residues 124 and 132 where we structurally move 2 Å along each of these modes. It can be observed that some modes show large changes between the chains A and C, and others between chains B and D, at the residues 124 and 132, and in some cases both of them.

Modes 66, 114, 135, 192 and 197 play a dominant role for the variations of the distances at residue Leu124. Modes 74, 80, and 123 play a similar role at the gating Tyr132. Modes 114 and 169 show a greater variation between the chains A and C, while modes 182 and 192 a greater variation between the chains B and D. These modes describe different structural motions like the kink of the transmembrane helices (modes 66, 74, 80 and 182, Movie S1), the swivel and uprising of the slides helices (modes 80, 169, 182 and 197, Movie S2), and the swing of cytoplasmic domain (mode 169, Movie S3).

### Molecular dynamics using excited normal modes (MDeNM)

The principle of the MDeNM^20^ is based on the propagation of the structural changes along a combination of low frequency normal mode vectors within the framework of molecular dynamics simulation by regularly injecting kinetic energy along the propagation direction. Thus, large conformational changes are promoted within a relatively short period of time compared to standard dynamics. In order to cover the whole normal mode space, the simulations are repeated for different linear combinations of selected NM vectors, here those that are relevant for the opening/closing of the channel. To have a limited number of nearly isotropically distributed combinations of modes relevant to the channel opening, we generated a large number of randomly distributed vectors, from which a subset was selected based on a rmsd filtering procedure. The filtering consists in a first step of structural displacement of the overall initial structure by 1 Å rmsd distance along the vectors, and in a second step in which a subset of modes is selected according to local rmsds in the channel region, higher than a threshold value. The vectors corresponding to this subset are considered in the MDeNM for their propagation. One advantage of having a filtering procedure is that we cover more completely the normal mode space generating a large set of different conformations. Another advantage of generating propagated structures along collective directions of motions is that local motions are coupled to global motions yielding energetically acceptable displaced structures. The period of time between two successive kinetic excitations is associated to the relaxation or adaptation of local motions to large-scale conformational changes. Every simulation pertaining to a given combination of NM vectors is independent of others, and is named here a “replica”. All the replica simulations are carried out in parallel by using a large number of CPU’s giving a significant computational speedup. In the present article, the channel region for which the rms filtering was performed covers the segment going from Met121 to Ala133 for all the four subunits. The rms threshold was fixed to 0.9 Å, yielding 62 collective motions to be excited. The kinetic excitation energy adopted corresponds to an increase of the temperature of the whole system of about 3 K, which represents a high kinetic input concentrated along a single direction at the start of the excitation. 10 kinetic excitations were performed along a given direction, a number sufficient for generating large conformational changes, and the period of time between subsequent excitations was about 1 ps. The parameters used in the MDeNM are given in Table S3.

Simulations were carried out with the CHARMM software using a specific script designed for MDeNM with the same MD parameters as used for the equilibration dynamics (the MDeNM script is available upon request).

### Relaxation of the excited MDeNM structures

The MDeNM simulations yielded 620 excited structures corresponding to the end of excitation periods. From this set we selected a lower number of structures that were almost equidistant between them, considering the local rmsds at the level of the channel so no biases are introduced and a given channel conformation is not being over represented. These structures were subjected to free MD simulations without any excitation to obtain a near Boltzmann equilibrium distribution from which open/closed state populations could be estimated and compared to the experimental data, and structural correlations be calculated.

To achieve this we applied the agglomerative hierarchical clustering tool of VMD^46^ with an rmsd threshold of 0.7 Å that yielded about 114 clusters. The nearest structure to the average structure in each cluster and the isolated structures not belonging to any of the clusters were considered as representative structures for starting free MD simulations (see Table S3 for more details). The duration of each individual MD was of 0.4 ns. We considered in our analysis only the last 0.3 ns of each trajectory devoid of prior MDeNM excitations. The simulations provided an ensemble of 34,086 structures that were used in our statistical analysis.

### Protein expression and purification

KirBac3.1 wild type was over-expressed and purified in *E.Coli* BL21 Codon Plus as outlined before^6^. Briefly, after cell disruption by French press, the protein was directly solubilized with 45 mM DM (Decyl β-D-maltopyranoside), centrifuged, and the supernatant was loaded onto a Co^2+^ affinity column. The protein was then purified promptly on a Superdex 200 column pre-equilibrated with 2 mM TriDM (n-Tridecyl β-maltoside) buffer. Concentrated (1–2.5 mg/ml) preparations of purified proteins (>95% purity, judged by SDS-PAGE) were stored at −80 °C in buffer containing 0.2 mM TriDM.

### Pepsin digestion

All protein digestions in solution were performed in an ice bath at 0 °C. Protease solutions were prepared in 500 mM glycine (pH 2.2). KirBac3.1 proteins were digested in the same buffer for 2–5 min using a protease/substrate ratio of 1:1 or 1:10 (wt/wt) for pepsin and nepenthesin, respectively, either in solution or immobilized on a resin. Increase in digestion time did not have any effect on the proteolysis.

### Hydrogen/deuterium exchange approach coupled to mass spectrometry (HDX-MS)

HDX-MS reactions were carried out on KirBac3.1 at a protein concentration of about 10 μM. The reaction was initiated by a 10x dilution of the protein samples (10 μL) into a deuterated buffer containing 50 mM KCl and 0.2 mM TriDM. Time course of the HDX was followed over a 20-min period by sequential withdrawing 120 μL of deuterated samples, which were immediately added to 26 μL of quenching buffer (8 M guanidium chloride, 500 mM glycine HCl, pH 2.2), rapidly mixed, and flash-frozen in liquid nitrogen.

### HPLC peptide separation

After protease digestion in solution or on column in an ice bath at 0 °C, peptides were loaded onto a peptideMicroTrap (Michrom Bioresources) column and washed with 0.03% TFA in water (HPLC solution A). They were then separated on a reversed-phase C12 column (1 × 50 mm, Jupiter; Phenomenex) using a linear gradient of 15–45% (vol/vol) of solution B (CH3CN 95% and TFA 0.03%) during 26 min. The column was connected to the electrospray source of mass spectrometers until 30% (vol/vol) B and then disconnected to avoid pollution of the electrospray source by the detergent. The valves, trap cartridge and column were cooled to 0 °C by immersion in an ice bath as previously described^22^.

### Mass spectrometric analyses of peptides

The tandem MS (mapping) analyses were performed on an ion trap mass spectrometer (Esquire 3000 + ; Bruker Daltonics) to enable the identification of the peptides after their separation on HPLC. Accurate mass measurements and the analysis of the local kinetics of deuteration were performed on a time-of-flight (TOF) mass spectrometer (6210; Agilent Technologies) equipped with an electrospray source. Each deuteration experiment was replicated in triplicate. Data were processed with MassHunter software (Agilent Technologies). Qualitative Analysis, and the deconvolution and calculation of the average masses were carried out in Magtran^47^.

### Electrophysiology

An Orbit mini was used (Nanion, Germany, horizontal planar lipid bilayer system), where two aqueous chambers (150 µL) are separated by a partition with a 100-µm hole where the lipid bilayer is formed by 1,2-diphytanoyl-sn-glycero-3-phosphocholine (DPhPC,15–30 pF). The lower chamber contained 150 mM KCl, 10 mM MOPS, pH 7.4. The upper chamber contained 150 mM KCl, 10 mM MOPS pH 8. 1 µL of purified KirBac3.1 (90 µg/mL) in DDM (n-DoDecyl-β-D-maltoside) detergent (0.015%) was added to the upper chamber to a preformed bilayer. Currents were recorded using Elements Data Reader (Nanion, Germany) and analyzed using Clampfit (Axon Instrument Inc, USA) software, sampled at 100 µs and filtered at 1.25 kHz.

## Supplementary information

Video S3.

Video S2.

Video S1.

Supplementary.
