# Supplementary material for: New Structural insights into Kir channel gating from molecular simulations, HDX-MS and functional studies
Source: Sci Rep. 2020 May 21;10:8392. doi: 10.1038/s41598-020-65246-z (PMC7242327; doi:10.1038/s41598-020-65246-z)
Supplement: Supplementary file 4 — Supplementary. [file 41598_2020_65246_MOESM4_ESM.docx]

**New Structural and functional insights into Kir**

**channel gating from molecular simulations,**

**HDX-MS and functional studies**

***Charline Fagnen^1,2^ Ludovic Bannwarth^1^, Iman Oubella ^1^, Eric Forest^3^, Rita De Zorzi^4^, Aline de Araujo^1,2^, Yasmina Mhoumadi^1,2^, Saïd Bendahhou^5^, David Perahia ^2^ and Catherine Vénien-Bryan^1^***

**^1^**Sorbonne Université, UMR 7590, CNRS, Muséum National d'Histoire Naturelle, Institut de Minéralogie, Physique des Matériaux et Cosmochimie, IMPMC, 75005 Paris, France.

^2^Laboratoire de Biologie et de Pharmacologie Appliquée, Ecole Normale Supérieure Paris-Saclay, Centre National de la Recherche Scientifique, 91190 Gif-sur-Yvette, Franc.

^3^University Grenoble Alpes, IBS, F-38044 Grenoble, France, CNRS, IBS, F-38044 Grenoble, France, CEA,IBS, F-38044 Grenoble, France.

^4^Department of Chemical and Pharmaceutical Sciences, University of Trieste, Via Licio Giorgeri 1, 34127, Trieste, Italy ^5^CNRS UMR7370, LP2M, Université Côte d’Azur, Nice Sophia-Antipolis, Faculté de Médecine, Nice, France.

**Introduction**


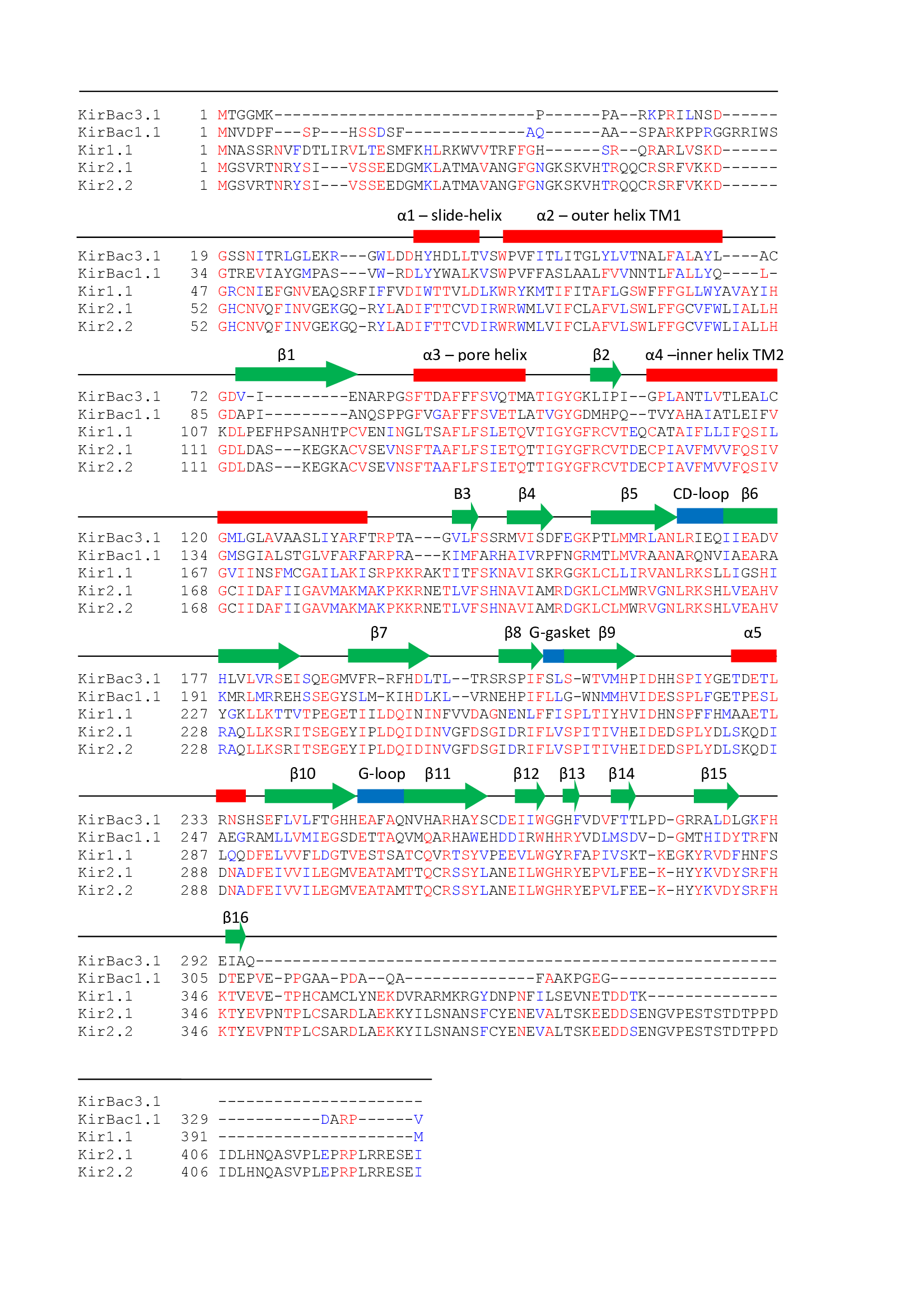


**Fig. S1 – Alignment of the sequences of the KirBac3.1, KirBac1.1, human Kir1, human Kir2.1 and chicken Kir2.2.** In red are represented the identical residues between the sequences and in blue the residues of similar types between the sequences. α-helix and β-strand are represented with red squares and green arrows, respectively. G-loop, CD-loop and G-gasket are noticed with a blue square.

**Results and Discussion**

**Selection of modes contributing to the gating**

Selection of a subset of most contributing normal modes to channel gating. The following criteria were used successively for the selection of relevant modes for gating: 1) Selection of modes showing local rmsd values of the channel greater than 1.25 Å; 2) Analysis of Cα-Cα distance variations between opposite chains (d(AC) and d(BD)) of Ala125, located in the middle of the channel. The ratio of both distances (d(AC)/d(BD)) is indicative of the channel deformation, whether circular or elliptical. We selected the modes contributing to the most prominent deformation patterns: 2a) modes with a ratio between 0.8 and 1.2 (mostly radial) and with local rmsds values larger than 1.75 Å, and 2b) modes outside this range (mostly elliptical); 3) To further decrease the number of modes, a selection criterion was applied at the constriction points (residues 124 and 132) by selecting the ten modes showing the largest d(AC) or d(BD) values. The application of these successive criteria resulted in the selection of 23 modes; 4) In a final step, redundant modes displaying similar backbone motions were identified and only those with the lower frequency were kept, considering that the side chain motions are already taken into account in the MD simulations.


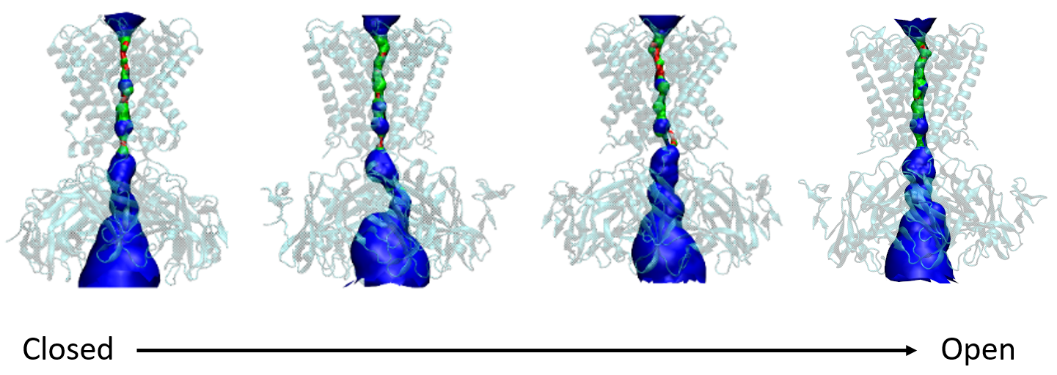


**Fig. S2 – Representation of the opening of the channel at four different states.** From left to right are represented the most closed structure, the most open of the closed structures, the most closed of the open structures and the most open structure. The channel is computed with the Hole software v2.2.005. Areas in red indicate where one water molecule cannot go through, green the areas where only one water molecule can go through and blue where more than one water molecule can go through. To select the represented structures among the closed states, we calculated the average of the distances between the chains A and C, and between B and D at the level of Leu124 and Tyr132. From left to right: the minimal value obtained corresponds to the most closed structure (1^st^ structure: radius at the level of Leu124: 1.04Å, radius at the level of Tyr132: 1.26Å), the maximal value represents the most open of the closed structures (2^nd^ structure: radius at the level of Leu124: 1.53Å, radius at the level of Tyr132: 1.56Å). To select the open structures, we select the structures showing the lowest (3^rd^ structure) and the highest values (4^th^ structure) for each constriction points between all opposite chains. Radii of the third structure from Hole are 1.79Å at the level of Leu124 and 1.77Å at the level of Tyr132. Radii of the fourth structure from Hole are 2.39Å at the level of Leu124 and 2.15Å at the level of Tyr132. Considering the K^+^ radius is 1.76Å, the open states show radii greater than the radius of the K^+^ ion and closed states show lower radii.


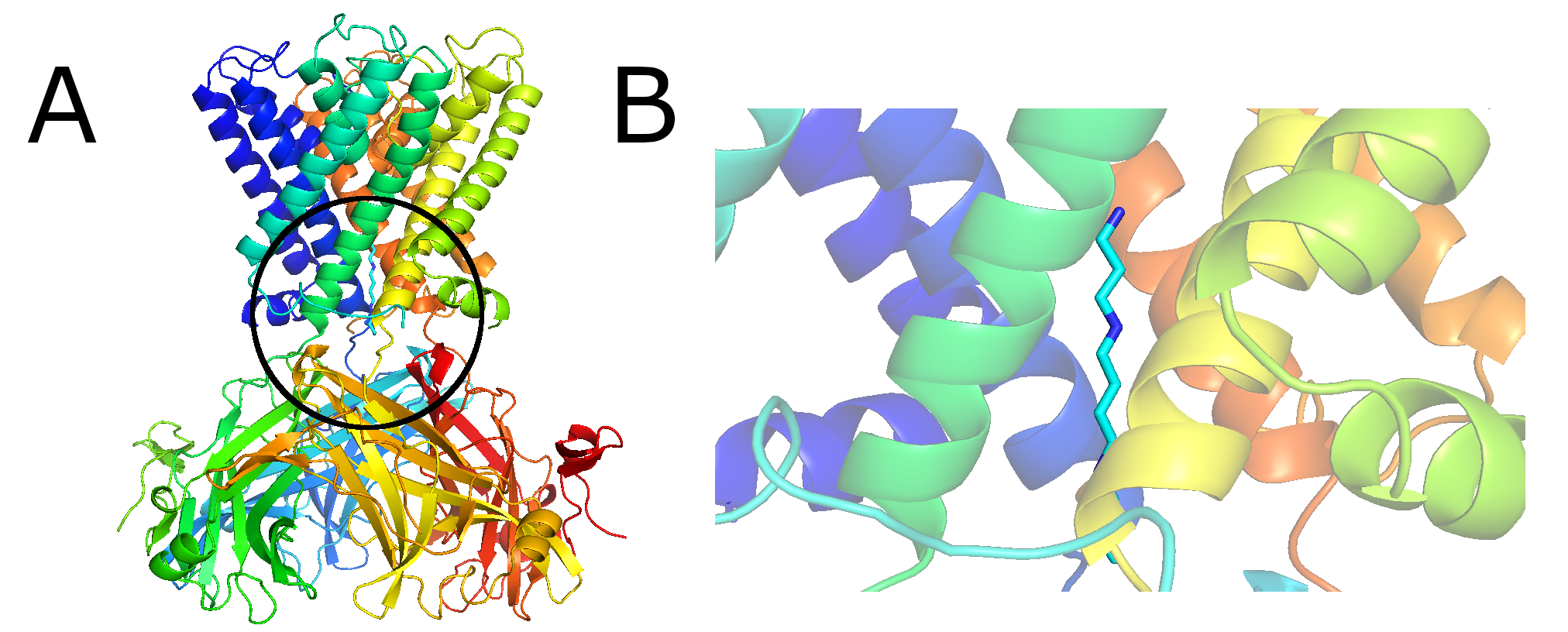


**Fig.S3 A:** Docking of the spermine molecule in one of our open structures of KirBac3.1 WT with the Autodock Vina software . B: enlarged structure. The spermine is well docked inside the channel and its location is similar to the one seen in the X-Ray structure of KirBac3.1 complexed with spermine, PDB 1XL6.


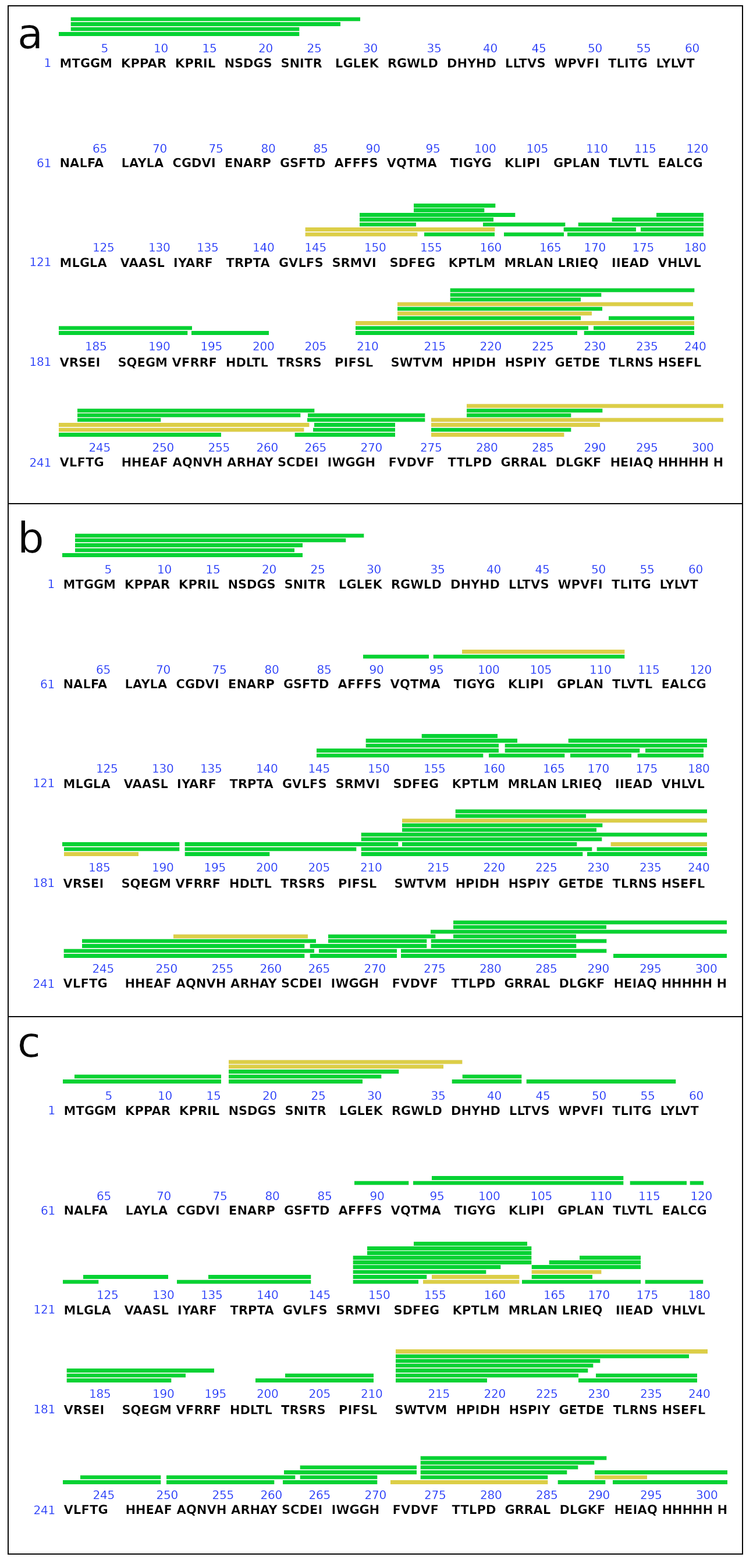


**Fig. S4 - Peptide mapping of KirBac3.1** using pepsin in solution (A), pepsin in column (B), and nepenthesin (C). The signal intensity of the peptides is shown in green (strong) and yellow (medium), see also Table. S1. We used nepenthesin because the coverage is larger.


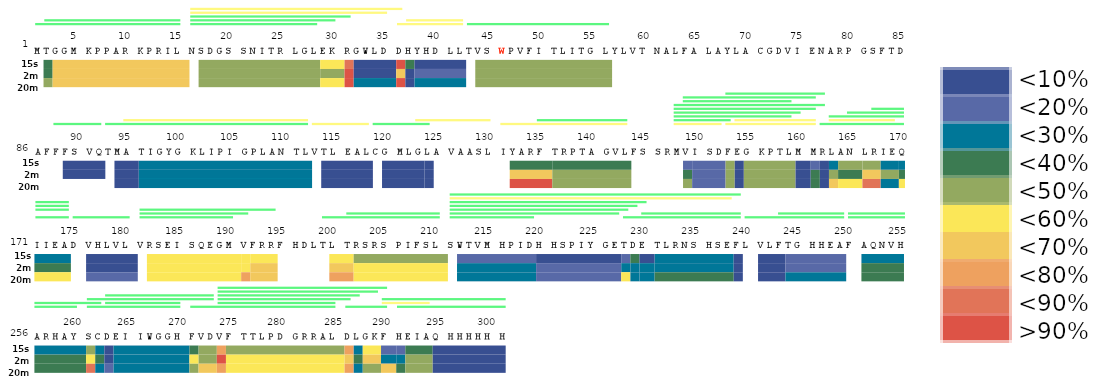


**Fig. S5 - Heat Map of the KirBac3.1.** Deuteration maps of KirBac3.1 peptides at different times up to 20 minutes deuteration. The color key indicates the HDX level.


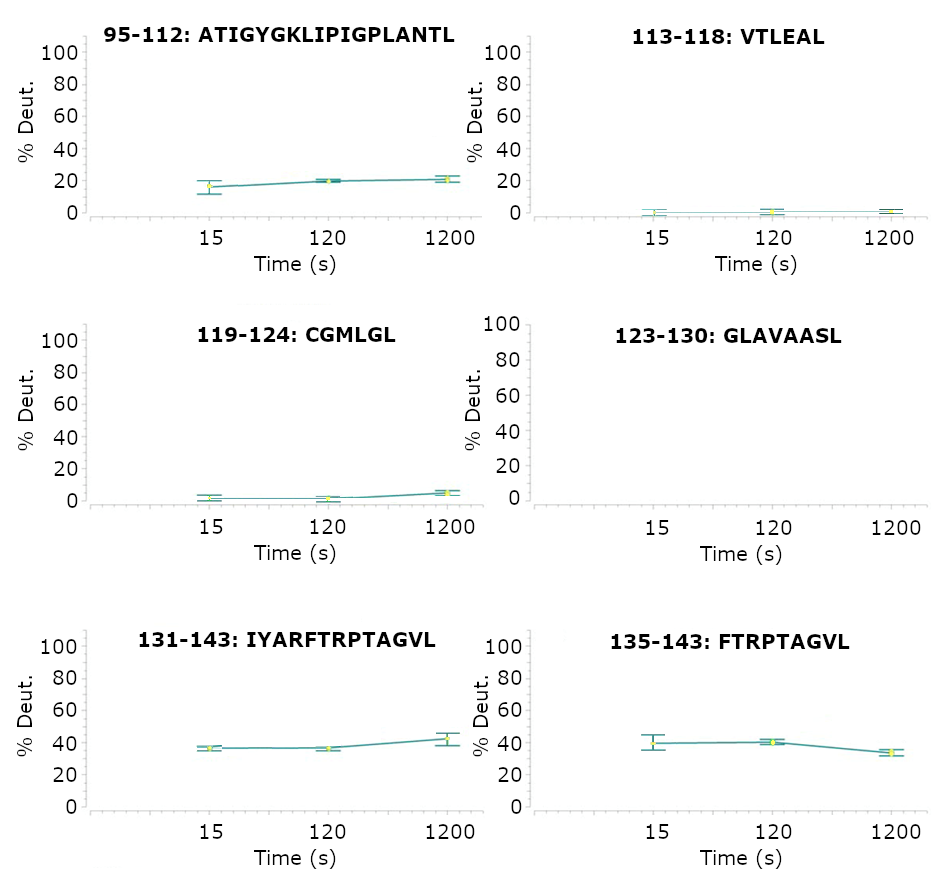


**Fig. S6** Percentage of deuteration versus time (15s, 120s and 1200s) for regions 95-112, 113-118, 119-124, 123-130, 131-143 and 135-143. The stretch from 113-130 is not covered.


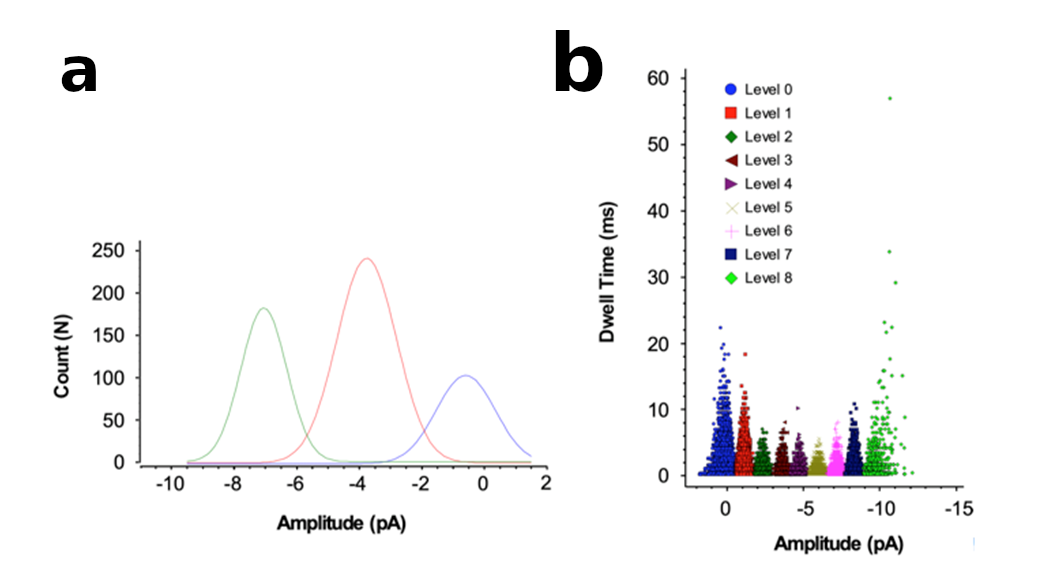


**Fig. S7** - Purified KirBac 3.1 channels are functionally active channels in planar lipid bilayers (a). Representative all point histograms of KirBac 3.1 WT from 6 minutes of continuous recording. Fits of Gaussian distributions of multiple histograms led to a single channel current level of 3.75 ± 0.027 pA at -80 mV (membrane bilayer contains two channels); (b) KirBac 3.1 channels has multiple subconductance states. Dwell time of the whole 6-min recording at -80 mV revealed channel subconductance levels. Fits of Gaussian distributions of multiple histograms showed current amplitudes (levels 1 to 8) at -1.02, -2.03, -3.6, -4.9, -6, -7.06, -8.1, and -9.5 pA.

**Discussion**


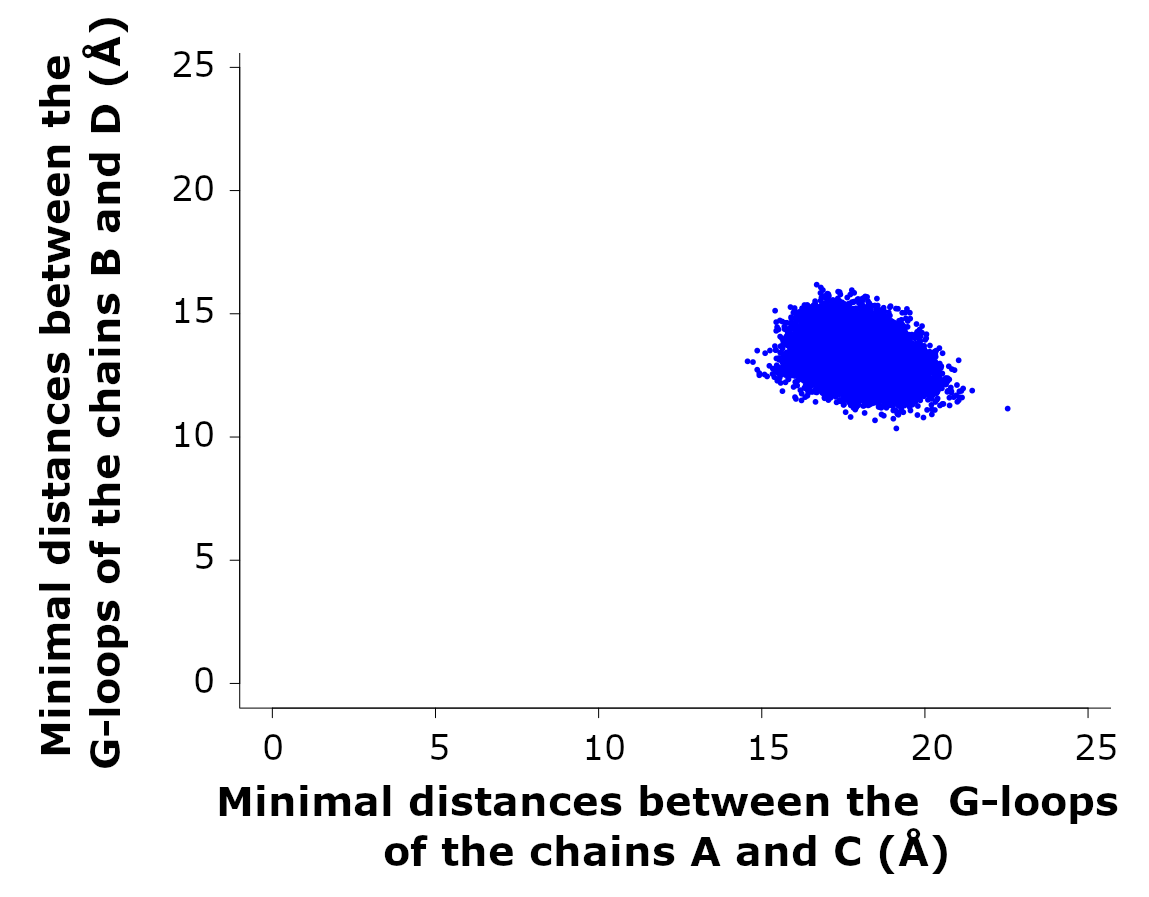


**Fig. S8 - Minimum distances of opposing G-loops.** Scatter plot of the minimal distances between the chains B and D and between the chains A and C at the level of the residues of the G-loop (residues Glu248 to Ala251).


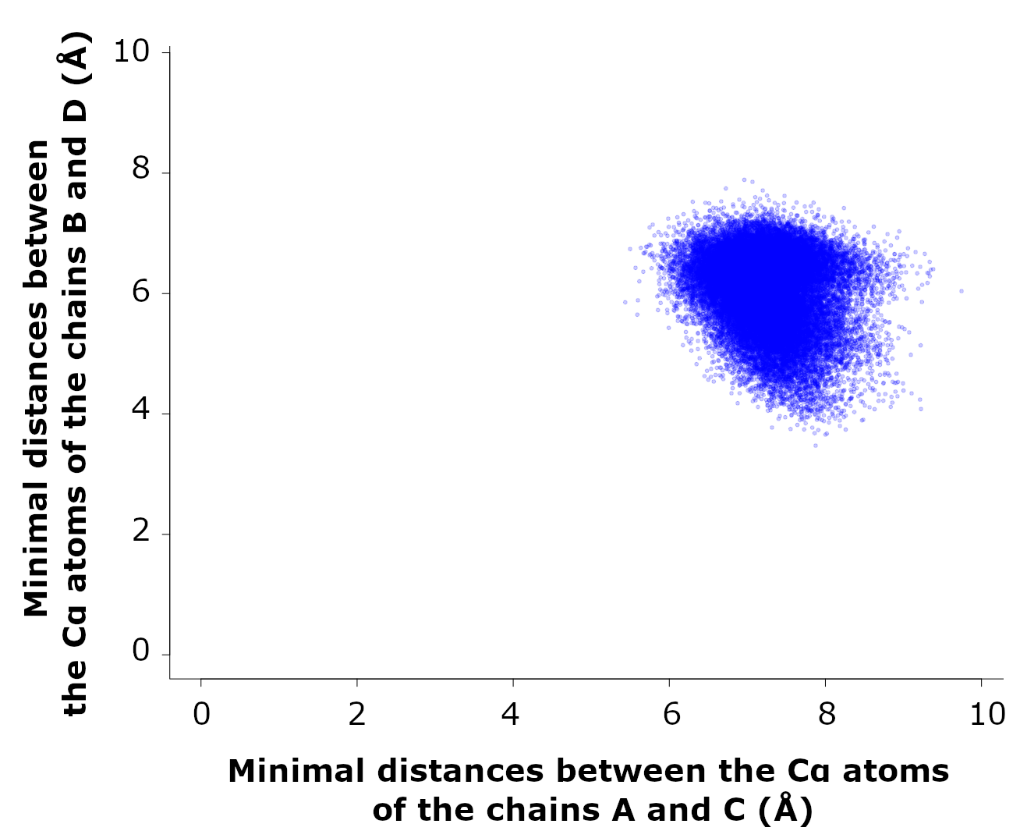


**Fig. S9 - Minimal distances at the level of opposite selective filter**. Scatter plot of the minimal distances between the Cα atoms of the chains B and D and between A and C at the level of the residues of the selective filter (residues Ile97 to Lys101).

**Material and methods**

**Structural determinants in relation to gating**

Several structural determinants related to gating were defined by analyzing the set of conformations generated by MDeNM and free MD simulations. These structural determinants are described in what follows:
*1) Minimal distances between the residues in the constriction points along the channel*

The closest (or minimal) distance between the atoms of the facing residues at the constriction points give a measure of the openness of the gate. As the same residues of different chains lie almost on the same plane parallel to the membrane, we considered only the projected distance on this plane. These variables are defined as *g124ac, g124bd, g132ac* and *g132bd*, and represent the minimal distance at the gating Leu124 between the chains A and C, and between the chains B and D, and at the gating Tyr132 between the chains A and C, and between the chains B and D, respectively.

*2) Kink angle of TM helices*
The kink of the inner TM helices is defined by the angle between the helical axes of the segments from Ala109 to Gly120 and from Gly120 to Phe135, as depicted in Fig 3a. For the outer helices, the axes of segments Cys71 to Leu56 and Leu56 to Trp46 were considered. Inner helix kink angles are named kia, kib, kic and kid corresponding to chains A, B, C, and D, respectively. Similarly, outer helix kink angles are named koa, kob, koc, and kod for chains A, B, C, and D, respectively
*3) Swing angles of the CTD*
The swing of the cytoplasmic domain of a given chain, related to its up/down motion with respect to the membrane, is described by a dihedral angle defined by four successive points linked by pseudo bonds (in green in Fig. 3b). The first two points are on the Z-axis (the symmetry axis of the molecule), corresponding to the projections of the Cβ atoms of Asp280 and of Phe275 on the axis, and the last two points are the Cα atoms of Phe275 and Asp280. The swing angles are designated as sa, sb, sc, and sd, corresponding to chains A, B, C and D, respectively.
*4) Twist angles of the CTD*
The twist motion of each chain around the symmetry axis is described by the variation of the dihedral angle defined by four successive points: the Cα atom of Leu108, the projection on the z-axis of atom Cγ of Leu108, the projection on the z-axis of atom Cγ2 of Ile266 and the Cα atom of Ile266, as depicted in Fig. 3b. The twist angles are specified by ta, tb, tc and td, referring to the corresponding chains.
*5) Swivel angle of the slide-helix*
The swivel motion of the slide helix describes how this helix displaces in a direction parallel to the membrane plane. It is described by a rotation about the symmetry axis. To calculate this parameter, we

considered the dihedral angle defined successfully by the Cα atom of Phe49, the projections on the z- axis of Cβ atoms of residues Phe49 and His37, and the Cα atom of His37. This dihedral angle is depicted in **Fig. 3b** in purple. The swivel angles of the slide helices is designated by *swia, swib, swic,* and *swid*, referring to the corresponding chains.

6) Uprising angle of the slide helix
The uprising angle of the slide helix describes the degree to which this helix approaches the plane of the membrane. It is defined by the angle between the Z-axis and the axis of the slide helix, from residue Thr43 to Asp35, as depicted in Fig. 3c. The uprising angles are specified by upa, upb, upc, and upd for the four chains.
7) Side chain conformational changes
Dihedral angles χ1 and χ2 of residues Leu124 and Tyr132 considered are defined between atoms N-Cα- Cβ-Cγ and Cα-Cβ-Cγ-Cδ1, the zero value corresponding to the cis configuration, and the positive value to an anti-clockwise rotation.
The correlations between the parameters were computed with the R software thanks to the module cor. This module estimates the Pearson coefficient between pairs of variables.

**Movie S1:** Kink of the inner and outer helices. The video represents the motion along the selected mode 182 of KirBac3.1. Each chain is represented in different color. A zoom on the chain A (blue) highlights the kink on the inner and outer helices at the level of Gly120 and Leu56 respectively.

**Movie S2:** Swivel and uprising motions of the slides helices. The video represents the motion along the selected mode 80 of KirBac3.1. Each chain is represented in different color. A zoom on the chain C (orange) highlights the swivel motion on the slide-helix, and another zoom on the chain A shows an uprising motion of the slide-helix.

**Movie S3:** Swing of the cytoplasmic domain. The video represents the motion along the selected mode 169 of KirBac3.1. Each chain is represented in different color. A zoom on the chain A (blue) highlights the swing motion of the cytoplasmic domain.

**Tables**

**Table S1**. **Sequence coverage for KirBac3.1** obtained in different digestion conditions and after deuteration.

| Protease | Condition | Coverage | Observation |
| --- | --- | --- | --- |
| Pepsin | In solution | 60% | Regions 29-143 and 201-208 not covered |
|  | column | 70% | Regions 29-88 and 113-143 not covered |
| Nepenthesin | column | 78% | Regions 57-87 (top half of TM1 + half of the pore helix), 143-147 (β3 + loop between β3-β4) and 195-203 (second half of β7) not covered |

**Table S2**. **Cα-Cα distance variations** (in Å) between opposite chains at the levels of the residues Leu124 and Tyr132 when displacing the energy minimized structure by 2 Å of rmsd along each of the 11 selected modes of WT.

| Modes | Leu124AC | Leu124BD | Tyr132AC | Tyr132BD |
| --- | --- | --- | --- | --- |
| 66 | 1.62 | 2.07 | 0.38 | 1.28 |
| 74 | 0.57 | 0.19 | 6.28 | 5.97 |
| 80 | 0.77 | 0.45 | 6.82 | 6.52 |
| 114 | 2.82 | 1.98 | 2.08 | 1.40 |
| 123 | 0.70 | 0.65 | 4.91 | 2.82 |
| 135 | 3.81 | 5.31 | 0.49 | 1.15 |
| 148 | 1.65 | 1.15 | 0.11 | 0.94 |
| 169 | 1.60 | 0.47 | 2.63 | 0.22 |
| 182 | 1.05 | 3.42 | 0.73 | 2.99 |
| 192 | 5.57 | 5.90 | 1.01 | 1.92 |
| 197 | 3.55 | 5.39 | 1.07 | 0.71 |

**Table S3. Parameters used in the MDeNM.**

| Properties | Parameters |
| --- | --- |
| Selected Modes | 66, 74, 80, 114, 123, 135, 148, 169, 182, 192, 197 |
| RMS cut-off MDeNM (Å) | 0.9 |
| Excitation temperature (K) | 3 |
| Number of excitations | 10 |
| Relaxation time (ps) | 1 |
| Number of replicas | 62 |
| RMS cut-off Clustering (Å) | 0.7 |
